# Genomics and clinical correlates of renal cell carcinoma

**DOI:** 10.1007/s00345-018-2429-x

**Published:** 2018-08-11

**Authors:** Thomas J. Mitchell, Sabrina H. Rossi, Tobias Klatte, Grant D. Stewart

**Affiliations:** 1 Cancer Genome Project, Wellcome Sanger Institute, Hinxton, CB10 1SA UK; 2 Academic Urology Group, Department of Surgery, University of Cambridge, Cambridge, CB2 0QQ UK; 3 Department of Urology, Cambridge University Hospitals NHS Foundation Trust, Hills Road, Cambridge, CB2 0QQ UK; 4 Department of Urology, Royal Bournemouth and Christchurch Hospitals NHS Foundation Trust, Bournemouth, BH7 7DW UK

**Keywords:** Renal cancer, Genomics, Mutations, Evolution, Prognosis, Therapy

## Abstract

**Purpose:**

Clear cell, papillary cell, and chromophobe renal cell carcinomas (RCCs) have now been well characterised thanks to large collaborative projects such as The Cancer Genome Atlas (TCGA). Not only has knowledge of the genomic landscape helped inform the development of new drugs, it also promises to fine tune prognostication.

**Methods:**

A literature review was performed summarising the current knowledge on the genetic basis of RCC.

**Results:**

The Von Hippel–Lindau (*VHL*) tumour suppressor gene undergoes bi-allelic knockout in the vast majority of clear cell RCCs. The next most prevalent aberrations include a cohort of chromatin-modifying genes with diverse roles including *PBRM1*, *SETD2*, *BAP1*, and *KMD5C*. The most common non-clear cell renal cancers have also undergone genomic profiling and are characterised by distinct genomic landscapes. Many recurrent mutations have prognostic value and show promise in aiding decisions regarding treatment stratification. Intra-tumour heterogeneity appears to hamper the clinical applicability of sparsely sampled tumours. Ways to abrogate heterogeneity will be required to optimise the genomic classification of tumours.

**Conclusion:**

The somatic mutational landscape of the more common renal cancers is well known. Correlation with outcome needs to be more comprehensively furnished, particularly for small renal masses, rarer non-clear cell renal cancers, and for all tumours undergoing targeted therapy.

## Introduction

In this review, we consider what is currently known of the genetic landscape of the commonest subtypes of renal cell cancer (RCC). A glossary has been provided to aid the understanding of specialist terminology (Table 1). Clear cell, papillary, and chromophobe cancers have now been well characterised thanks to the development of sequencing technologies (Table 2) and large collaborative projects such as The Cancer Genome Atlas (TCGA). Not only has knowledge of the genomic landscape helped inform the development of new drugs, this understanding also promises to improve risk stratification of tumours and to determine their sensitivity to systemic therapies. We shall consider each subtype in turn describing genes and pathways of oncogenesis and how these relate to prognosis and treatment response. We finish by discussing limitations of these metrics before widespread clinical applicability may be adopted.

**Table 1:** Glossary of terms used in this manuscript

Term	Definition
5′UTR	The 5′ untranslated region is located downstream of where transcription begins but upstream of the first protein-coding region
A:T-to-T:A transversions	The substitution of a purine for a pyrimidine or vice versa in DNA. This changes the base from adenine (A) to thymine (T) (or vice versa)
Allele	Different versions of the same gene are called alleles. Humans have two alleles at each genetic locus, with one allele inherited from each parent
Arm-level loss	Loss of genetic material from the end of a chromosome (telomere) to the centre (centromere)
Autosome	Any chromosome that is not a sex chromosome (i.e., in chromosomes 1–22 in humans)
Bi-allelic knockout	Inactivation of both copies of a gene
Chromatin	Chromatin is a set of molecules found in cells whose primary function is to package DNA into a more compact structure
Clonal expansion	In cancer evolution, clonal expansion is the production of daughter cells with the same genetic makeup as the original cell
Convergent evolution	A process where independent clones evolve with similar traits, likely as a result of pressures to survive and grow within the tumour microenvironment
Copy number aberration	Gain or loss of part of a chromosome
Driver gene	A driver gene is one whose mutations increase the oncogenic potential of a tumour
Epigenetic	Epigenetic refers to non-genetic influences on gene expression
Epithelial to mesenchyme transition	Epithelial–mesenchymal transition describes the process by which epithelial cells lose their cell polarity and cell–cell adhesion, and gain migratory and invasive properties
Focal deletions	Deletion of genomic material within a chromosome spanning in general less than 5 million base pairs
Gene fusion	The result of a re-arrangement between different parts of the genome that aligns two genes
Germline mutation	A germline mutation is one present in the germ cells, i.e., can be passed onto offspring
GWAS	A genome-wide association study (GWAS), is an observational study of a genome-wide set of genetic variants in different individuals to see if any variant is associated with a trait
Haploinsufficiency	Presence of only one functional copy of a gene (see mono-allelic inactivation)
Loci	Loci refers to a position within the genome
Loss of heterozygosity	The loss of one allele of a genetic locus
Methyltransferase	Methyltransferases are a large group of enzymes that all methylate their substrates. In genetics, this affects gene transcription
Mono-allelic inactivation	Inactivation of one copy of a gene. We are born with two copies of all genes (aside from those on the sex chromosomes)
Mutational burden	The total number of mutations present within a cell or tumour
Mutational signature	For the context of this paper, mutational signatures relate to the effect of mutational processes such as age or smoking on the specific types of point mutation seen in the tumour. For instance, signature 1 is found in all cancer types and is associated with age at diagnosis. Signature 2 is attributed to the AID/APOBEC family of cytidine deaminases. See https://cancer.sanger.ac.uk/cosmic/signatures for details
Non-synonymous mutation	A mutation in the protein-coding part of the genome that results in a change in the resulting amino acid sequence
Point mutations	Alteration of a single base in the genome to an alternative base
Proto-oncogene	A normal gene which, when altered by mutation, becomes an oncogene—one that can contribute to cancer
Somatic mutation	Mutations that are acquired during the lifetime of an individual, i.e., are not inherited
Splice-site variants	A genetic alteration in the DNA sequence that occurs at the boundary of a protein-coding and non-coding region. This change can, therefore, alter the protein-coding sequence
Stochastically	A random probability distribution that cannot be precisely predicted
Structural variants	Variation in the structure of a chromosome. This encompasses many changes including the abnormal joining of different chromosomal regions and copy number aberrations
Telomeres	Repetitive genetic sequence at the ends of a chromosome that protect against degradation or fusion with other chromosomes
Trisomy	A trisomy is where there are three copies of a particular chromosome, instead of the normal two (in humans)
Ubiquitination	The addition of ubiquitin to a substrate protein which affects their subsequent use, interaction, localisation, or breakdown
Warburg-like metabolic shift	This is a phenomenon, whereby cells can produce additional energy through increased oxygen-dependent glycolysis followed by lactic acid fermentation

**Table 2:** Overview of the development of sequencing technologies that have enabled the understanding of the genetic component of cancer development

Methods	Explanation
Cytogenetics	These are methods used to study the structure and function of chromosomes. In cancer, they commonly refer to methods such as karyotyping, fluorescent in situ hybridisation (FISH), and comparative genomic hybridization (CGH), and give an overview of which areas of the chromosome may have been lost or gained during oncogenesis
Polymerase chain reaction (PCR)	The amplification of a few copies of a short region of DNA, generating thousands to millions of copies of this sequence. This allows the detection of mutations within the amplified sequence
Next generation sequencing	Next generation sequencing (NGS), massively parallel or deep sequencing are related terms that describe a DNA sequencing technology that can sequence an entire human genome within a single day. This is a catch-all term used to describe a number of different sequencing technologies such as Illumina (Solexa), Roche 454, Ion torrent or SOLiD sequencing

DNA sequencing technologies allow us to “read” DNA. By comparing the sequence of DNA in cancer compared to normal cells, we can identify changes that might be driving the growth of cancer. Comparisons between patients with cancer and those without may also reveal the presence of inherited mutations

## Methods

A non-systematic literature search was conducted using Medline, updated to May 2018. The reference lists of selected manuscripts were checked manually for eligible articles. The most relevant articles summarising existing knowledge on RCC genomics, including tumour cell evolution and progression, were selected for this review. Recurrent aberrations have been defined as those with false discovery rates of < 0.1 and reported in multiple studies in the literature. For prognostic markers, those events that are significant after multiple hypothesis testing have been included (adjusted *p* value < 0.1). The commonly accepted significance threshold (*p* < 5 × 10^−8^) has been used for genome-wide association studies (GWAS).

## Results

### Clear cell renal cell carcinoma

#### Epidemiology and genetics

The mutational landscape of clear cell RCC (ccRCC) has been defined most recently through several large-scale whole genome-sequencing studies [1–4]. These studies reveal that recurrent somatic mutations occur in only a handful of genes, with an overall mutational burden of roughly 1–2 per Mb. In addition, there are only a small number of recurrent copy number aberrations and rare gene fusions. Some insights into clinical risk factors and their genomic correlates have been made. These include patient age, with mutational burden correlating strongly with age via the predominance of the clock-like mutational signature in these genomes [5]. The higher incidence of ccRCC in male patients may partially be accounted by mono-allelic inactivation of the chromatin remodelling gene, *KDM5C* on the X chromosome [6]. No tobacco-specific mutational process has been detected despite the strong clinical risk factor [5]. The high frequency of A:T-to-T:A transversions consistent with mutational damage as a result of aristolochic acid exposure was detected via sequencing of patients from Eastern Europe [1] and has directly influenced primary prevention strategies.

Genetic risk factors are known to play a role in sporadic RCC development [7, 8]. Patients who have at least one first degree relative with RCC are at an increased risk of developing the disease (OR 1.4, 95% CI 0.71–2.76) [9]. The first renal large-scale GWAS in Europe revealed susceptibility loci at 2p21 and 11q13.3 [10]. The two correlated variants on 2p21 map to *EPAS1,* a transcription factor previously implicated in RCC, whereas the variant on 11q13.3 contains no characterised genes. An additional susceptibility locus on 12p11.23 was later discovered containing two variants in the *ITPR2* gene, though direct functional evidence between ITPR2 and oncogenesis is lacking [11]. Subsequently, a locus on intron 2 of the *ZEB2* gene was discovered which may play a role in decreasing regulation of epithelial to mesenchymal transition [12].

Most recently, a variant on 8q24.21 was discovered via interrogation of GWAS from an Icelandic population [13], prior to discovery of an additional seven new loci in the largest such GWAS study to date [14]. More comprehensive details of these loci are shown in Table 3.

**Table 3:** Summary of the inherited genetic locations believed to be associated with the development of RCC

Name	Chromosome band	Possible gene	Putative mechanism	References
rs11894252	2p21	*EPAS1*	Transcription factor encoding hypoxia-inducible-factor-2 alpha	[10]
rs7579899	2p21	*EPAS1*	Transcription factor encoding hypoxia-inducible-factor-2 alpha	[10]
rs7105934	11q13.3	–	–	[10]
rs718314	12p11.23	*ITPR*	Possibly through obesity related effects	[11]
rs1049380	12p11.23	*ITPR*	Possibly through obesity related effects	[11]
rs12105918	2q22.3	*ZEB2*	Regulation of the epithelial to mesenchyme transition	[12]
rs35252396	8q24.21	–	Within potential regulatory region associated with other solid cancers	[13]
rs4381241	1p32.3	*FAF1*	Facilitates increased expression of genes driving cell proliferation	[14]
rs67311347	3p22.1	*CTNNB1*	Facilitates increased expression of genes driving cell proliferation	[14]
rs10936602	3q26.2			[14]
rs2241261	8p21.3	*GFRA2*	Activation of the RET tyrosine kinase receptor	[14]
rs11813268	10q24	*OBFC1*	Regulator of telomere length	[14]
rs74911261	11q22.3	*KDELC2*	Functional disruption in endoplasmic reticulum	[14]
rs4903064	14q24.2	*DPF3*	Chromatin remodelling	[14]

#### Somatic mutations

##### VHL

**Fig. 1:**
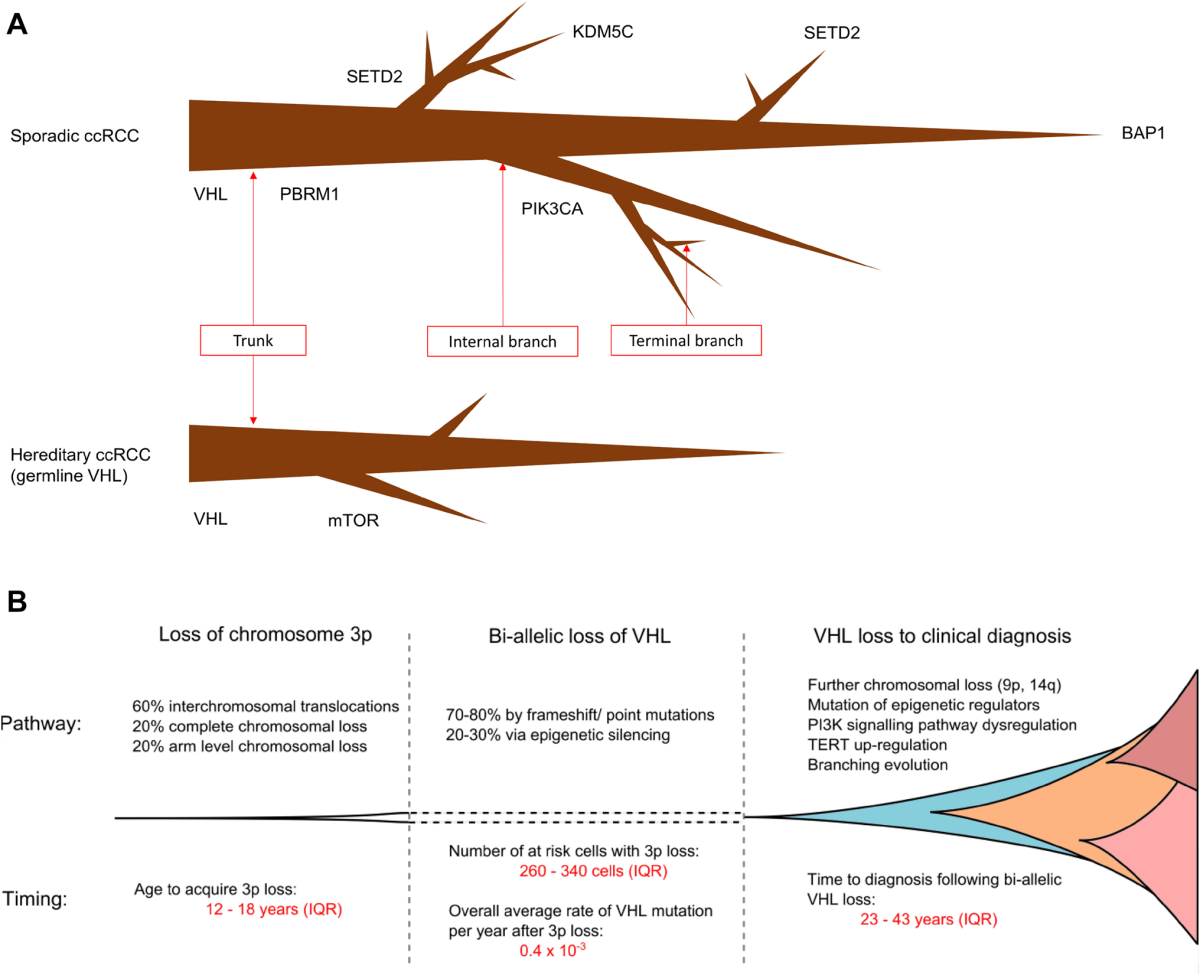
Schematic depicting **a**—trunk-branch model of tumour development (based on [50]) and **b**—evolution of ccRCC (based on [16])

**Fig. 2:**
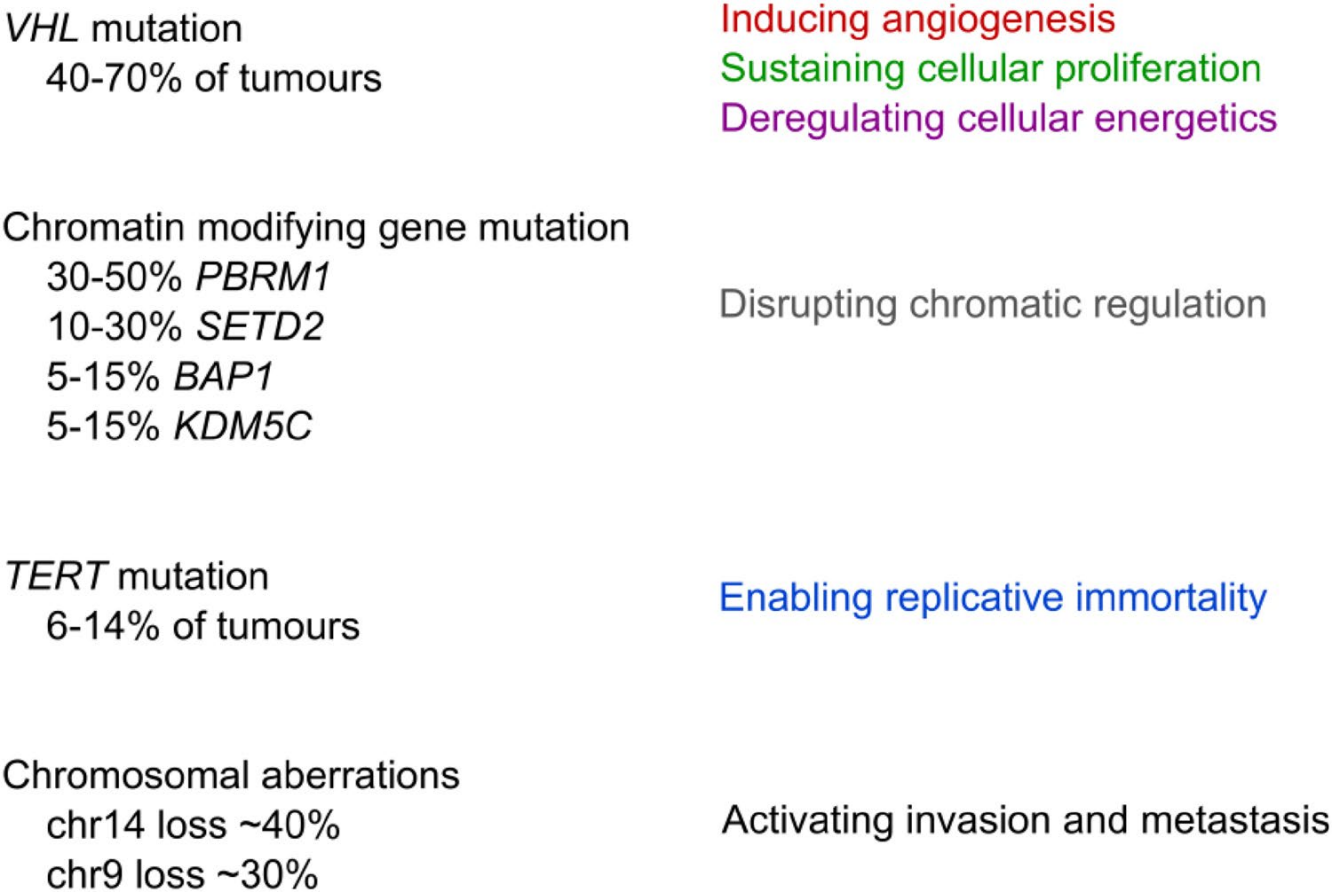
Mutation frequencies of the most commonly mutated genes in ccRCC and their effect as described by the Hallmarks of Cancer [64]

The Von Hippel–Lindau (*VHL*) tumour suppressor gene, located at 3p25, undergoes bi-allelic knockout in the majority of ccRCCs [15]. Haploinsufficiency of *VHL* occurs via arm-level loss of chromosome 3p in over 90% of tumours. Astonishingly, this event appears to occur in a handful of cells in childhood or late adolescence, often many decades prior to diagnosis [16] (Fig. 1). The second copy of *VHL* is lost, usually much later in life, by either non-synonymous mutation or epigenetic down-regulation [1–4, 16]. *VHL* inactivation prevents the ubiquitination of hypoxia-inducible factor (HIF) for degradation. Upregulation of HIF is advantageous to tumour cell survival due to increased expression levels of angiogenic factors, lower rates of apoptosis, and higher rates of cellular proliferation (Fig. 2). The ubiquitous nature of upregulated HIF pathways and, therefore, neo-angiogenesis has provided the rationale for treatment with vascular endothelial growth factor (VEGF) inhibitors. Perhaps, unsurprisingly, given its ubiquitous role, no consistent relationship between *VHL* status and clinical outcome has been found [17, 18].

##### Chromatin-modifying genes

A cohort of chromatin-modifying genes with diverse roles including *PBRM1, SETD2, BAP1,* and *KMD5C* constitutes the next most prevalent somatic mutations. The first three of these genes are also co-located with *VHL* on chromosome 3p, meaning that after 3p loss, any further non-synonymous mutation will result in complete inactivation of these haploinsufficient genes. *PBRM1,* a methyltransferase is the second most commonly mutated gene in ccRCC, found in 30–50% of tumours [1, 2]. *PBRM1*’s inactivation could lead to loss of DNA methylation via reduction of H3K36me3 [2]. *SETD2* is mutated in 10–30% of ccRCCs [1, 19–21]. *SETD2*’s intracellular roles are numerous, including the regulation of transcription elongation, RNA processing, and double-stranded DNA break repair [22] that may then activate the p53-mediated checkpoint in the absence of specific *p53* mutations [23]. *BAP1,* a histone deubiquitinase, is mutated in up to 5–15% of ccRCCs [1–3, 24, 25]. *BAP1*’s other roles include control of cellular proliferation and regulation of DNA damage repair [24].

Due to their prevalence, *PBRM1, SETD2,* and *BAP1* have all been investigated as prognostic markers and for possible treatment stratification. A retrospective, validated analysis found that tumours with *BAP1* mutations conferred a worse prognosis, higher grade, and worse overall survival when compared to those with *PBRM1* mutations or when compared to those without *BAP1* mutations [2, 24–26]. The presence of *BAP1* and *PBRM1* mutants appeared anti-correlated, though when co-existing, their presence conferred the worst overall survival. Similarly, the presence of *SETD2* confers worse overall survival by a hazard ratio of 1.7 [26].

Genomic profiling of tumours from patients with ccRCC is beginning to illustrate how the presence of mutations in chromatin-modifying genes may aid systemic treatment stratification. For instance, in the RECORD-3 trial [27], different sequences of everolimus (an mTOR inhibitor) and sunitinib (a VEGF inhibitor) appeared to affect progression free survival in metastatic patients according to *PBRM1* and *BAP1* status. Immuno-oncological agents are now showing increasing promise in metastatic ccRCC settings, where *PBRM1* mutations appear to confer clinical benefit after treatment with these agents [28].

##### TERT

Somatic mutations have been detected within the core promoter [27, 29, 30] and 5’UTR [16] of telomerase reverse transcriptase (*TERT*) in 6–14% of ccRCCs. Their functional corollary appears to include the lengthening of telomeres [16]. Furthermore, the presence of *TERT* promoter mutations has been shown to decrease cancer-specific survival [29] and increased disease stage [30].

##### PTEN- and mTOR-signalling pathway

The *PTEN* gene undergoes both recurrent point mutations (2–12% of samples) and focal deletions (approximately 7% of samples) in ccRCC [2, 3, 27]. The gene encodes a protein and phospholipid phosphatase that controls the balance between cell proliferation, apoptosis, and migration via the PI(3)K/AKT/MTOR pathway. Specific mutations may act in a dominant negative manner, implying that bi-allelic knockout is not always required to suppress function [31]. An interrogation of the TCGA data set revealed that bi-allelic loss of *PTEN* was uncommon but conferred worse overall survival. Mono-allelic loss was also associated with higher stage and histological grade [32]. Tumours with mutant *PTEN* status showed a non-significant increase in rates of progression when compared to non-*PTEN* mutant tumours after either VEGFR or MTOR inhibition in metastatic patients in the RECORD-3 trial [27].

The PI3K-AKT-mTOR signalling axis is directly augmented via *MTOR* mutations, observed in 4–9% of ccRCC neoplasms [1, 3]. Numerous other genes are involved in the mTOR pathway including *PTEN,* whose importance is discussed above. Other members of the pathway, such as TSC1/TSC2/PIK3CA, are infrequently mutated. Although FGFR4 undergoes copy gain as part of an arm-level gain of the long-arm of chromosome 5 in approximately 50% of ccRCC, a direct causal link between this event and PI3K-AKT-mTOR has not yet been conclusively found. The PI3K–mTOR pathway is an important growth factor-signalling cascade that alters cellular metabolism. It is, therefore, an attractive target for systemic therapies via compounds collectively named rapalogs that bind to FKBP12 to inhibit the PI3K–AKT–mTOR pathway via abrogated mTORC1 kinase activity [33]. There is some evidence that tumours with mutations in *MTOR*/*TSC1/2* have a better response to rapalogs, although statistical significance has not been reached [27, 34, 35].

##### TP53

*TP53* appears relatively infrequently mutated in ccRCC (2–9% of tumours) [2, 3]. However, aberrations in genes involved in the *P53* pathway are relatively common implying that the p53 pathway and cell-cycle checkpoint inhibition play significant roles in ccRCC [3, 23]. *TP53* appears to be more frequently mutated in metastases [36] and on survival analysis, confers a worse cancer-specific survival than any other single mutation [37].

##### Structural variants

Structural variants promote oncogenesis by altering the number of copies of genes in the genome or re-arranging the order of the genome such that either new genes are formed or the expression of a gene is altered. Aside from translocation renal cell carcinoma, gene fusions are uncommon in renal cell cancer. Large-scale copy number variations are common, however, including the almost ubiquitous heterozygous loss of the short arm of chromosome 3. The next most common aberrations are: gain of chromosome 5 (~ 60% of samples) and loss of chromosome 14 (~ 40% of samples), loss of chromosomes 6q, 8p, and 9p, and gain of chromosome 7 (~ 30% of samples) [1–4, 16, 37, 38]. Multiple authors have investigated copy number aberrations as potential biomarkers, mainly using array comparative genomic hybridisation and cytogenetic studies. Although many of these aberrations have been shown to predict prognosis [38–43], only few were repeatedly validated on multivariable analyses. Due to most copy number alterations covering large segments of the genome, it is difficult to uncover the mechanism by which the change confers oncogenic advantage. Some interesting and notable genes within these regions include *CDKN2A* on chromosome 9p, which has been shown to modulate VEGF expression via its interaction with HIF-1alpha, encoded by *HIF1A* on 14q [44].

#### Immunotherapy and mutational burden

Targeted immunotherapy, for instance, in the form of programmed death 1 (PD-1) checkpoint inhibition or cytotoxic T-lymphocyte associated antigen 4 (CTLA-4) inhibition is being increasingly used in both first- and second-line therapies [45]. Prediction of favourable response has not been correlated with PD-1 ligand biomarker expression [46]. In bladder cancer amongst others [47], mutational burden as a surrogate for neoantigen levels has been associated with enhanced response to targeted immunotherapy. Although the total mutational burden is relatively low in renal cancer, it may be a useful adjunct for response prediction to novel immunotherapeutic agents. Recently, a small study correlated total mutational burden with response to targeted immunotherapy in RCC [48]. In this study, estimated tumour mutational burden was similar in those with progressive disease and clinical benefit (10 vs. 11, *p* = 0.8), as was the duration of therapy in patients with high- and low-tumour mutational burden (71 vs. 70 days, *p* = 0.39). However, this study was fairly small (*n* = 31) and included both patients with ccRCC and non-clear cell RCC who received several different targeted immunotherapies.

#### Heterogeneity

Intratumoural heterogeneity in ccRCC is well understood through bulk tissue DNA sequencing with branching evolution occurring more commonly and earlier than other cancer types [4, 49]. It dominates the evolutionary landscape at the genomic, transcriptomic, and proteomic levels [50]. Phylogenetic analyses led to the trunk-branch model of RCC development (Fig. 1). Somatic mutations that are found in all sampled tumour regions present in the trunk of the phylogenetic tree, including *VHL* mutations and 3p loss [51, 52]. This finding supports the Knudson two-hit hypothesis [53], where two ‘hits’ (i.e., bi-allelic inactivation of *VHL*) are required for clonal expansion to yield a clone large enough to stochastically acquire independent branches. Less prevalent mutations appear more commonly on branches with the same gene sometimes mutated on different branches in a fashion consistent with convergent evolution [4, 52]. One direct corollary of these findings is that the estimation of driver gene prevalence based on single regional sequencing significantly under-estimates the true tumour-based estimation. Increased driver prevalence was seen, particularly in *PBRM1, BAP1, TP53, PTEN, PIK3CA,* and *TSC2*. Incomplete molecular profiling from single biopsies may hinder accurate prognosis and response to therapy. Extensive multi-regional sequencing appears to demarcate tumour behaviour according to evolutionary subtypes [4].

Clearly, the development of methods to infer tumour behaviour without resorting to exhaustive spatial tissue sampling is vital. Current estimates of the sampling density required to adequately represent tissue biology lie between 3 and 8 [4, 51], making these methods impractical in clinical practice. Alternatively, tumour behaviour and the Darwinian phylogeny may be predicted from other methods of molecular profiling such as transcriptomic analysis or functional imaging.

### Papillary renal cell carcinoma

Papillary RCC (pRCC) represents approximately 20% of all kidney cancers and accumulates mutations at a similar rate to ccRCC [54], again with a predominance of a clock-like process. Signature 2, associated with APOBEC family of cytidine deaminases is the next most common signature. Classified as either type I or type II in roughly equal proportions, pRCC occurs either sporadically or as an inherited form [7]. In general, type I cancers are often multifocal and confer a better prognosis than the more aggressive and typically unifocal type II cancers [55]. Although classified as separate entities, types I and II pRCCs share many molecular features, including chromatin modifications seen in ccRCC [56]. Some of the shared genomic features, such as gene fusions involving *TFE3* or *TFEB*, are present in approximately 10% of samples and show no particular disposition to type I or type II cancers [56]. The functional implication of these events remains unclear [57]. Due to the molecular overlap between both types and the fact that prognostic significance of types I vs. II was not confirmed on multivariable analyses, the clinical utility of papillary type has been questioned [55].

#### Type I

Hereditary papillary renal cancer (HPRC) predisposes to type I pRCC via autosomal dominant inheritance of a mutation in the *MET* proto-oncogene [7]. Increased *MET* mRNA expression is commonly observed in the sporadic form of the disease [56]. This increased expression is potentially directly driven by whole chromosomal copy number aberrations, in particular trisomy 7 which is present in the majority of type I pRCC tumours. In addition, approximately fifteen per cent of sporadic cases harbour activating mutations in the tyrosine kinase domain or contain splice-site variants [56, 58]. The MET pathway interacts with other key oncological pathways such as RAS and PIK3 causes increased angiogenesis and increased cell dissociation and is, therefore, the subject of interest for targeted inhibition. A multicentre phase II study investigated foretinib, an inhibitor of MET, VEGFR2, RON, and AXL tyrosine kinase in sporadic and HPRC-associated papillary RCC [59]. Although overall response rates were moderate, half of patients with germline *MET* mutations had a partial response. Unfortunately, no other pathological or molecular review was undertaken, but improved treatment stratification by type I or *MET* mutational status shows promise.

Additional genes recurrently mutated in type I pRCC include *KDM6A*, *SMARCB1* and *NFE2L2* [56]. Despite widening the net to discover other candidate driver mutations through known-cancer associated genes, one-third of tumours had no clearly discernible driver, other than trisomy of broad copy number alterations, most commonly chromosome 7.

#### Type II

The inherited form of the more aggressive, type II pRCC tumours is caused by germline mutation of the gene encoding fumarate hydratase (FH) [7]. Sporadic *FH* mutations are rarely found; however, mutations of genes in the downstream NRF2–antioxidant response element (ARE) pathway such as *NFE2L2* are recurrently detected.

*CDKN2A* alterations are present in 25% of type II pRCC tumours when loss of heterogeneity, promoter hypermethylation, and somatic mutations are considered together [56]. Increased expression of cell-cycle related genes was seen, most likely via retinoblastoma protein. The presence of *CDKN2A* alterations was also adversely associated survival in univariate analysis of the whole cohort and when limited to the more aggressive type II phenotype [56].

A small subset of type II pRCC; CpG island methylator phenotype (CIMP) had universal hypermethylation of the *CDKN2A* promoter and also a high prevalence of germline or somatic mutations in *FH* [56]. These tumours expressed increased levels of hypoxia-related genes and evidence of a Warburg-like metabolic shift. These effects underpin the rationale to trial agents such as bevacizumab (VEGF inhibition) and erlotinib (TKI), in the phase II setting (NCT01130519).

The chromatin-modifying genes *SETD2*, *BAP1*, and *PBRM1* are recurrently mutated in the absence of consistent loss of heterozygosity or promoter hypermethylation [56].

### Chromophobe renal cell carcinoma

Chromophobe RCC (chRCC) accounts for 5% of renal carcinomas, but this figure is higher amongst young women. It is relatively indolent, although sarcomatoid differentiation renders it highly aggressive [55]. These tumours derived from the distal nephron accumulate mutations at a low rate (~ 0.4 per Mb) [54]. The most characteristic feature is extensive whole chromosomal loss of heterozygosity involving chromosomes 1, 2, 6, 10, 13, 17, and 21 [60]. The *TP53* and *PTEN* genes were recurrently mutated almost exclusively in classic (i.e., non-eosinophilic) variants [60, 61]. Recurrent aberrations in the *TERT* gene were also detected with some harbouring the canonical 228T mutations, but mainly via structural variants that correlated strongly with increased TERT expression [60]. The eosinophilic subtype, describing an eosinophilic cytoplasm with densely packed mitochondria, harboured cases that were devoid of copy number aberrations and some that were enriched for the mitochondrial *MT*-*ND5* gene mutations [60, 61]. The causal mechanism between this mutation and the histopathological phenotype has not yet been ascertained.

There are little data on genomic correlates with patient outcomes. Sun et al. analysed 66 patients from the TCGA database. *TP53* mutations were found in 33% of tumours, while loss of *HNF1B* was seen in 88%. Prevalence of both *TP53* mutations and loss of *HNF1b* increased with tumour stage and were linked with poor survival [62]. Casuscelli et al. [63] studied genomic outcome correlates of 79 chRCCs of all stages. *TP53* mutations (58%), *PTEN* mutations (24%), and imbalanced chromosome duplication (duplication of ≥ 3 chromosomes, 25%) were enriched in patients with metastatic disease. While each feature was associated with inferior survival, the combination of all three changes yielded the worst prognosis.

### Oncocytoma

Oncocytomas are benign tumours that share many features with eosinophilic chRCCs, including derivation from the distal tubule and recurrent mutations in mitochondria-encoded proteins [61]. Type 2 oncocytomas also contains recurrent whole chromosome losses that resemble those seen in eosinophilic chRCC [61]. The absence of *PT53* mutations and activation of the p53 pathway in oncocytomas highlights p53 as a barrier to oncocytoma progression.

## Conclusions

Scientific literature provides a detailed view of the genomic landscapes for each of the more common renal cancers. The genomic archaeology of clear cell tumours is particularly well characterised through exhaustive multi-regional sequencing. Through this knowledge, there is the potential to better stratify the risk of progression and survival for kidney cancer. Emerging evidence is showing that the presence or the absence of certain mutations may relate to therapeutic sensitivity or resistance. There are a number of gaps in our knowledge; these particularly relate to the behaviour of small renal masses, rarer subtypes of renal cell cancer, and the response of tumours to newer targeted agents.

## References

[1] Scelo G, Riazalhosseini Y, Greger L, Letourneau L, Gonzalez-Porta M, Wozniak MB, Bourgey M, Harnden P, Egevad L, Jackson SM, Karimzadeh M, Arseneault M, Lepage P, How-Kit A, Daunay A, Renault V, Blanche H, Tubacher E, Sehmoun J, Viksna J, Celms E, Opmanis M, Zarins A, Vasudev NS, Seywright M, Abedi-Ardekani B, Carreira C, Selby PJ, Cartledge JJ, Byrnes G, Zavadil J, Su J, Holcatova I, Brisuda A, Zaridze D, Moukeria A, Foretova L, Navratilova M, Mates D, Jinga V, Artemov A, Nedoluzhko A, Mazur A, Rastorguev S, Boulygina E, Heath S, Gut M, Bihoreau MT, Lechner D, Foglio M, Gut IG, Skryabin K, Prokhortchouk E, Cambon-Thomsen A, Rung J, Bourque G, Brennan P, Tost J, Banks RE, Brazma A, Lathrop GM (2014). Variation in genomic landscape of clear cell renal cell carcinoma across Europe. Nat Commun.

[2] Cancer Genome Atlas Research N (2013). Comprehensive molecular characterization of clear cell renal cell carcinoma. Nature.

[3] Sato Y, Yoshizato T, Shiraishi Y, Maekawa S, Okuno Y, Kamura T, Shimamura T, Sato-Otsubo A, Nagae G, Suzuki H, Nagata Y, Yoshida K, Kon A, Suzuki Y, Chiba K, Tanaka H, Niida A, Fujimoto A, Tsunoda T, Morikawa T, Maeda D, Kume H, Sugano S, Fukayama M, Aburatani H, Sanada M, Miyano S, Homma Y, Ogawa S (2013). Integrated molecular analysis of clear-cell renal cell carcinoma. Nat Genet.

[4] Turajlic S, Xu H, Litchfield K, Rowan A, Horswell S, Chambers T, O’Brien T, Lopez JI, Watkins TBK, Nicol D, Stares M, Challacombe B, Hazell S, Chandra A, Mitchell TJ, Au L, Eichler-Jonsson C, Jabbar F, Soultati A, Chowdhury S, Rudman S, Lynch J, Fernando A, Stamp G, Nye E, Stewart A, Xing W, Smith JC, Escudero M, Huffman A, Matthews N, Elgar G, Phillimore B, Costa M, Begum S, Ward S, Salm M, Boeing S, Fisher R, Spain L, Navas C, Gronroos E, Hobor S, Sharma S, Aurangzeb I, Lall S, Polson A, Varia M, Horsfield C, Fotiadis N, Pickering L, Schwarz RF, Silva B, Herrero J, Luscombe NM, Jamal-Hanjani M, Rosenthal R, Birkbak NJ, Wilson GA, Pipek O, Ribli D, Krzystanek M, Csabai I, Szallasi Z, Gore M, McGranahan N, Van Loo P, Campbell P, Larkin J, Swanton C, Consortium TRR (2018) Deterministic evolutionary trajectories influence primary tumor growth: TRACERx renal. Cell 173(3):595–610 e511. 10.1016/j.cell.2018.03.043 10.1016/j.cell.2018.03.043 PMC5938372 29656894

[5] Alexandrov LB, Jones PH, Wedge DC, Sale JE, Campbell PJ, Nik-Zainal S, Stratton MR (2015). Clock-like mutational processes in human somatic cells. Nat Genet.

[6] Ricketts CJ, Linehan WM (2015). Gender specific mutation incidence and survival associations in clear cell renal cell carcinoma (CCRCC). PLoS One.

[7] Maher ER (2018). Hereditary renal cell carcinoma syndromes: diagnosis, surveillance and management. World J Urol.

[8] Rossi SH, Klatte T, Usher-Smith J, Stewart GD (2018). Epidemiology and screening for renal cancer. World J Urol.

[9] Hung RJ, Moore L, Boffetta P, Feng BJ, Toro JR, Rothman N, Zaridze D, Navratilova M, Bencko V, Janout V, Kollarova H, Szeszenia-Dabrowska N, Mates D, Chow WH, Brennan P (2007). Family history and the risk of kidney cancer: a multicenter case-control study in Central Europe. Cancer Epidemiol Biomark Prev.

[10] Purdue MP, Johansson M, Zelenika D, Toro JR, Scelo G, Moore LE, Prokhortchouk E, Wu X, Kiemeney LA, Gaborieau V, Jacobs KB, Chow WH, Zaridze D, Matveev V, Lubinski J, Trubicka J, Szeszenia-Dabrowska N, Lissowska J, Rudnai P, Fabianova E, Bucur A, Bencko V, Foretova L, Janout V, Boffetta P, Colt JS, Davis FG, Schwartz KL, Banks RE, Selby PJ, Harnden P, Berg CD, Hsing AW, Grubb RL, Boeing H, Vineis P, Clavel-Chapelon F, Palli D, Tumino R, Krogh V, Panico S, Duell EJ, Quiros JR, Sanchez MJ, Navarro C, Ardanaz E, Dorronsoro M, Khaw KT, Allen NE, Bueno-de-Mesquita HB, Peeters PH, Trichopoulos D, Linseisen J, Ljungberg B, Overvad K, Tjonneland A, Romieu I, Riboli E, Mukeria A, Shangina O, Stevens VL, Thun MJ, Diver WR, Gapstur SM, Pharoah PD, Easton DF, Albanes D, Weinstein SJ, Virtamo J, Vatten L, Hveem K, Njolstad I, Tell GS, Stoltenberg C, Kumar R, Koppova K, Cussenot O, Benhamou S, Oosterwijk E, Vermeulen SH, Aben KK, van der Marel SL, Ye Y, Wood CG, Pu X, Mazur AM, Boulygina ES, Chekanov NN, Foglio M, Lechner D, Gut I, Heath S, Blanche H, Hutchinson A, Thomas G, Wang Z, Yeager M, Fraumeni JF, Skryabin KG, McKay JD, Rothman N, Chanock SJ, Lathrop M, Brennan P (2011). Genome-wide association study of renal cell carcinoma identifies two susceptibility loci on 2p21 and 11q13.3. Nat Genet.

[11] Wu X, Scelo G, Purdue MP, Rothman N, Johansson M, Ye Y, Wang Z, Zelenika D, Moore LE, Wood CG, Prokhortchouk E, Gaborieau V, Jacobs KB, Chow WH, Toro JR, Zaridze D, Lin J, Lubinski J, Trubicka J, Szeszenia-Dabrowska N, Lissowska J, Rudnai P, Fabianova E, Mates D, Jinga V, Bencko V, Slamova A, Holcatova I, Navratilova M, Janout V, Boffetta P, Colt JS, Davis FG, Schwartz KL, Banks RE, Selby PJ, Harnden P, Berg CD, Hsing AW, Grubb RL, Boeing H, Vineis P, Clavel-Chapelon F, Palli D, Tumino R, Krogh V, Panico S, Duell EJ, Quiros JR, Sanchez MJ, Navarro C, Ardanaz E, Dorronsoro M, Khaw KT, Allen NE, Bueno-de-Mesquita HB, Peeters PH, Trichopoulos D, Linseisen J, Ljungberg B, Overvad K, Tjonneland A, Romieu I, Riboli E, Stevens VL, Thun MJ, Diver WR, Gapstur SM, Pharoah PD, Easton DF, Albanes D, Virtamo J, Vatten L, Hveem K, Fletcher T, Koppova K, Cussenot O, Cancel-Tassin G, Benhamou S, Hildebrandt MA, Pu X, Foglio M, Lechner D, Hutchinson A, Yeager M, Fraumeni JF, Lathrop M, Skryabin KG, McKay JD, Gu J, Brennan P, Chanock SJ (2012). A genome-wide association study identifies a novel susceptibility locus for renal cell carcinoma on 12p11.23. Hum Mol Genet.

[12] Henrion M, Frampton M, Scelo G, Purdue M, Ye Y, Broderick P, Ritchie A, Kaplan R, Meade A, McKay J, Johansson M, Lathrop M, Larkin J, Rothman N, Wang Z, Chow WH, Stevens VL, Ryan Diver W, Gapstur SM, Albanes D, Virtamo J, Wu X, Brennan P, Chanock S, Eisen T, Houlston RS (2013). Common variation at 2q22.3 (ZEB2) influences the risk of renal cancer. Hum Mol Genet.

[13] Gudmundsson J, Sulem P, Gudbjartsson DF, Masson G, Petursdottir V, Hardarson S, Gudjonsson SA, Johannsdottir H, Helgadottir HT, Stacey SN, Magnusson OT, Helgason H, Panadero A, van der Zanden LF, Aben KK, Vermeulen SH, Oosterwijk E, Kong A, Mayordomo JI, Sverrisdottir A, Jonsson E, Gudbjartsson T, Einarsson GV, Kiemeney LA, Thorsteinsdottir U, Rafnar T, Stefansson K (2013) A common variant at 8q24.21 is associated with renal cell cancer. Nat Commun 4:2776. 10.1038/ncomms3776 10.1038/ncomms3776 24220699

[14] Scelo G, Purdue MP, Brown KM, Johansson M, Wang Z, Eckel-Passow JE, Ye Y, Hofmann JN, Choi J, Foll M, Gaborieau V, Machiela MJ, Colli LM, Li P, Sampson JN, Abedi-Ardekani B, Besse C, Blanche H, Boland A, Burdette L, Chabrier A, Durand G, Le Calvez-Kelm F, Prokhortchouk E, Robinot N, Skryabin KG, Wozniak MB, Yeager M, Basta-Jovanovic G, Dzamic Z, Foretova L, Holcatova I, Janout V, Mates D, Mukeriya A, Rascu S, Zaridze D, Bencko V, Cybulski C, Fabianova E, Jinga V, Lissowska J, Lubinski J, Navratilova M, Rudnai P, Szeszenia-Dabrowska N, Benhamou S, Cancel-Tassin G, Cussenot O, Baglietto L, Boeing H, Khaw KT, Weiderpass E, Ljungberg B, Sitaram RT, Bruinsma F, Jordan SJ, Severi G, Winship I, Hveem K, Vatten LJ, Fletcher T, Koppova K, Larsson SC, Wolk A, Banks RE, Selby PJ, Easton DF, Pharoah P, Andreotti G, Freeman LEB, Koutros S, Albanes D, Mannisto S, Weinstein S, Clark PE, Edwards TL, Lipworth L, Gapstur SM, Stevens VL, Carol H, Freedman ML, Pomerantz MM, Cho E, Kraft P, Preston MA, Wilson KM, Michael Gaziano J, Sesso HD, Black A, Freedman ND, Huang WY, Anema JG, Kahnoski RJ, Lane BR, Noyes SL, Petillo D, Teh BT, Peters U, White E, Anderson GL, Johnson L, Luo J, Buring J, Lee IM, Chow WH, Moore LE, Wood C, Eisen T, Henrion M, Larkin J, Barman P, Leibovich BC, Choueiri TK, Mark Lathrop G, Rothman N, Deleuze JF, McKay JD, Parker AS, Wu X, Houlston RS, Brennan P, Chanock SJ (2017). Genome-wide association study identifies multiple risk loci for renal cell carcinoma. Nat Commun.

[15] Gnarra JR, Tory K, Weng Y, Schmidt L, Wei MH, Li H, Latif F, Liu S, Chen F, Duh FM (1994). Mutations of the VHL tumour suppressor gene in renal carcinoma. Nat Genet.

[16] Mitchell TJ, Turajlic S, Rowan A, Nicol D, Farmery JHR, O’Brien T, Martincorena I, Tarpey P, Angelopoulos N, Yates LR, Butler AP, Raine K, Stewart GD, Challacombe B, Fernando A, Lopez JI, Hazell S, Chandra A, Chowdhury S, Rudman S, Soultati A, Stamp G, Fotiadis N, Pickering L, Au L, Spain L, Lynch J, Stares M, Teague J, Maura F, Wedge DC, Horswell S, Chambers T, Litchfield K, Xu H, Stewart A, Elaidi R, Oudard S, McGranahan N, Csabai I, Gore M, Futreal PA, Larkin J, Lynch AG, Szallasi Z, Swanton C, Campbell PJ, Consortium TRR (2018) Timing the landmark events in the evolution of clear cell renal cell cancer: TRACERx renal. Cell 173 (3):611–623 e617. 10.1016/j.cell.2018.02.020 10.1016/j.cell.2018.02.020 PMC5927631 29656891

[17] Smits KM, Schouten LJ, van Dijk BA, Hulsbergen-van de Kaa CA, Wouters KA, Oosterwijk E, van Engeland M, van den Brandt PA (2008). Genetic and epigenetic alterations in the von hippel-lindau gene: the influence on renal cancer prognosis. Clin Cancer Res.

[18] Patard JJ, Fergelot P, Karakiewicz PI, Klatte T, Trinh QD, Rioux-Leclercq N, Said JW, Belldegrun AS, Pantuck AJ (2008). Low CAIX expression and absence of VHL gene mutation are associated with tumor aggressiveness and poor survival of clear cell renal cell carcinoma. Int J Cancer.

[19] Dalgliesh GL, Furge K, Greenman C, Chen L, Bignell G, Butler A, Davies H, Edkins S, Hardy C, Latimer C, Teague J, Andrews J, Barthorpe S, Beare D, Buck G, Campbell PJ, Forbes S, Jia M, Jones D, Knott H, Kok CY, Lau KW, Leroy C, Lin ML, McBride DJ, Maddison M, Maguire S, McLay K, Menzies A, Mironenko T, Mulderrig L, Mudie L, O’Meara S, Pleasance E, Rajasingham A, Shepherd R, Smith R, Stebbings L, Stephens P, Tang G, Tarpey PS, Turrell K, Dykema KJ, Khoo SK, Petillo D, Wondergem B, Anema J, Kahnoski RJ, Teh BT, Stratton MR, Futreal PA (2010). Systematic sequencing of renal carcinoma reveals inactivation of histone modifying genes. Nature.

[20] Varela I, Tarpey P, Raine K, Huang D, Ong CK, Stephens P, Davies H, Jones D, Lin ML, Teague J, Bignell G, Butler A, Cho J, Dalgliesh GL, Galappaththige D, Greenman C, Hardy C, Jia M, Latimer C, Lau KW, Marshall J, McLaren S, Menzies A, Mudie L, Stebbings L, Largaespada DA, Wessels LF, Richard S, Kahnoski RJ, Anema J, Tuveson DA, Perez-Mancera PA, Mustonen V, Fischer A, Adams DJ, Rust A, Chan-on W, Subimerb C, Dykema K, Furge K, Campbell PJ, Teh BT, Stratton MR, Futreal PA (2011). Exome sequencing identifies frequent mutation of the SWI/SNF complex gene PBRM1 in renal carcinoma. Nature.

[21] Guo G, Gui Y, Gao S, Tang A, Hu X, Huang Y, Jia W, Li Z, He M, Sun L, Song P, Sun X, Zhao X, Yang S, Liang C, Wan S, Zhou F, Chen C, Zhu J, Li X, Jian M, Zhou L, Ye R, Huang P, Chen J, Jiang T, Liu X, Wang Y, Zou J, Jiang Z, Wu R, Wu S, Fan F, Zhang Z, Liu L, Yang R, Liu X, Wu H, Yin W, Zhao X, Liu Y, Peng H, Jiang B, Feng Q, Li C, Xie J, Lu J, Kristiansen K, Li Y, Zhang X, Li S, Wang J, Yang H, Cai Z, Wang J (2012). Frequent mutations of genes encoding ubiquitin-mediated proteolysis pathway components in clear cell renal cell carcinoma. Nat Genet.

[22] Li J, Duns G, Westers H, Sijmons R, van den Berg A, Kok K (2016). SETD2: an epigenetic modifier with tumor suppressor functionality. Oncotarget.

[23] Carvalho S, Vitor AC, Sridhara SC, Martins FB, Raposo AC, Desterro JM, Ferreira J, de Almeida SF (2014). SETD2 is required for DNA double-strand break repair and activation of the p53-mediated checkpoint. eLife.

[24] Pena-Llopis S, Vega-Rubin-de-Celis S, Liao A, Leng N, Pavia-Jimenez A, Wang S, Yamasaki T, Zhrebker L, Sivanand S, Spence P, Kinch L, Hambuch T, Jain S, Lotan Y, Margulis V, Sagalowsky AI, Summerour PB, Kabbani W, Wong SW, Grishin N, Laurent M, Xie XJ, Haudenschild CD, Ross MT, Bentley DR, Kapur P, Brugarolas J (2012). BAP1 loss defines a new class of renal cell carcinoma. Nat Genet.

[25] Kapur P, Pena-Llopis S, Christie A, Zhrebker L, Pavia-Jimenez A, Rathmell WK, Xie XJ, Brugarolas J (2013). Effects on survival of BAP1 and PBRM1 mutations in sporadic clear-cell renal-cell carcinoma: a retrospective analysis with independent validation. Lancet Oncol.

[26] Hakimi AA, Ostrovnaya I, Reva B, Schultz N, Chen YB, Gonen M, Liu H, Takeda S, Voss MH, Tickoo SK, Reuter VE, Russo P, Cheng EH, Sander C, Motzer RJ, Hsieh JJ, cc RCCCGARNi (2013). Adverse outcomes in clear cell renal cell carcinoma with mutations of 3p21 epigenetic regulators BAP1 and SETD2: a report by MSKCC and the KIRC TCGA research network. Clin Cancer Res.

[27] Hsieh JJ, Chen D, Wang PI, Marker M, Redzematovic A, Chen YB, Selcuklu SD, Weinhold N, Bouvier N, Huberman KH, Bhanot U, Chevinsky MS, Patel P, Pinciroli P, Won HH, You D, Viale A, Lee W, Hakimi AA, Berger MF, Socci ND, Cheng EH, Knox J, Voss MH, Voi M, Motzer RJ (2016). Genomic biomarkers of a randomized trial comparing first-line everolimus and sunitinib in patients with metastatic renal cell carcinoma. Eur Urol.

[28] Miao D, Margolis CA, Gao W, Voss MH, Li W, Martini DJ, Norton C, Bosse D, Wankowicz SM, Cullen D, Horak C, Wind-Rotolo M, Tracy A, Giannakis M, Hodi FS, Drake CG, Ball MW, Allaf ME, Snyder A, Hellmann MD, Ho T, Motzer RJ, Signoretti S, Kaelin WG, Choueiri TK, Van Allen EM (2018). Genomic correlates of response to immune checkpoint therapies in clear cell renal cell carcinoma. Science.

[29] Hosen I, Rachakonda PS, Heidenreich B, Sitaram RT, Ljungberg B, Roos G, Hemminki K, Kumar R (2015). TERT promoter mutations in clear cell renal cell carcinoma. Int J Cancer.

[30] Wang K, Liu T, Liu L, Liu J, Liu C, Wang C, Ge N, Ren H, Yan K, Hu S, Bjorkholm M, Fan Y, Xu D (2014). TERT promoter mutations in renal cell carcinomas and upper tract urothelial carcinomas. Oncotarget.

[31] Papa A, Wan L, Bonora M, Salmena L, Song MS, Hobbs RM, Lunardi A, Webster K, Ng C, Newton RH, Knoblauch N, Guarnerio J, Ito K, Turka LA, Beck AH, Pinton P, Bronson RT, Wei W, Pandolfi PP (2014). Cancer-associated PTEN mutants act in a dominant-negative manner to suppress PTEN protein function. Cell.

[32] Lee HJ, Lee HY, Lee JH, Lee H, Kang G, Song JS, Kang J, Kim JH (2014). Prognostic significance of biallelic loss of PTEN in clear cell renal cell carcinoma. J Urol.

[33] Kang SA, Pacold ME, Cervantes CL, Lim D, Lou HJ, Ottina K, Gray NS, Turk BE, Yaffe MB, Sabatini DM (2013). mTORC1 phosphorylation sites encode their sensitivity to starvation and rapamycin. Science.

[34] Kwiatkowski DJ, Choueiri TK, Fay AP, Rini BI, Thorner AR, de Velasco G, Tyburczy ME, Hamieh L, Albiges L, Agarwal N, Ho TH, Song J, Pignon JC, Barrios PM, Michaelson MD, Van Allen EM, Krajewski KM, Porta C, Pal SK, Bellmunt J, McDermott DF, Heng DY, Gray KP, Signoretti S (2016). Mutations in TSC1, TSC2, and MTOR are associated with response to rapalogs in patients with metastatic renal cell carcinoma. Clin Cancer Res.

[35] Voss MH, Hakimi AA, Pham CG, Brannon AR, Chen YB, Cunha LF, Akin O, Liu H, Takeda S, Scott SN, Socci ND, Viale A, Schultz N, Sander C, Reuter VE, Russo P, Cheng EH, Motzer RJ, Berger MF, Hsieh JJ (2014). Tumor genetic analyses of patients with metastatic renal cell carcinoma and extended benefit from mTOR inhibitor therapy. Clin Cancer Res.

[36] de Velasco G, Wankowicz SA, Madison R, Ali SM, Norton C, Duquette A, Ross JS, Bosse D, Lalani AA, Miller VA, Stephens PJ, Young L, Hakimi AA, Signoretti S, Pal SK, Choueiri TK (2018). Targeted genomic landscape of metastases compared to primary tumours in clear cell metastatic renal cell carcinoma. Br J Cancer.

[37] Gulati S, Martinez P, Joshi T, Birkbak NJ, Santos CR, Rowan AJ, Pickering L, Gore M, Larkin J, Szallasi Z, Bates PA, Swanton C, Gerlinger M (2014). Systematic evaluation of the prognostic impact and intratumour heterogeneity of clear cell renal cell carcinoma biomarkers. Eur Urol.

[38] Turajlic S, Xu H, Litchfield K, Rowan A, Chambers T, Lopez JI, Nicol D, O’Brien T, Larkin J, Horswell S, Stares M, Au L, Jamal-Hanjani M, Challacombe B, Chandra A, Hazell S, Eichler-Jonsson C, Soultati A, Chowdhury S, Rudman S, Lynch J, Fernando A, Stamp G, Nye E, Jabbar F, Spain L, Lall S, Guarch R, Falzon M, Proctor I, Pickering L, Gore M, Watkins TBK, Ward S, Stewart A, DiNatale R, Becerra MF, Reznik E, Hsieh JJ, Richmond TA, Mayhew GF, Hill SM, McNally CD, Jones C, Rosenbaum H, Stanislaw S, Burgess DL, Alexander NR, Swanton C, Peace, Consortium TRR (2018) Tracking cancer evolution reveals constrained routes to metastases: TRACERx renal. Cell 173(3):581–594 e512. 10.1016/j.cell.2018.03.057 10.1016/j.cell.2018.03.057 PMC5938365 29656895

[39] El-Mokadem I, Fitzpatrick J, Bondad J, Rauchhaus P, Cunningham J, Pratt N, Fleming S, Nabi G (2014). Chromosome 9p deletion in clear cell renal cell carcinoma predicts recurrence and survival following surgery. Br J Cancer.

[40] Shen C, Beroukhim R, Schumacher SE, Zhou J, Chang M, Signoretti S, Kaelin WG (2011). Genetic and functional studies implicate HIF1alpha as a 14q kidney cancer suppressor gene. Cancer Discov.

[41] Kroeger N, Klatte T, Chamie K, Rao PN, Birkhauser FD, Sonn GA, Riss J, Kabbinavar FF, Belldegrun AS, Pantuck AJ (2013). Deletions of chromosomes 3p and 14q molecularly subclassify clear cell renal cell carcinoma. Cancer.

[42] Klatte T, Kroeger N, Rampersaud EN, Birkhauser FD, Logan JE, Sonn G, Riss J, Rao PN, Kabbinavar FF, Belldegrun AS, Pantuck AJ (2012). Gain of chromosome 8q is associated with metastases and poor survival of patients with clear cell renal cell carcinoma. Cancer.

[43] Klatte T, Rao PN, de Martino M, LaRochelle J, Shuch B, Zomorodian N, Said J, Kabbinavar FF, Belldegrun AS, Pantuck AJ (2009). Cytogenetic profile predicts prognosis of patients with clear cell renal cell carcinoma. J Clin Oncol.

[44] Zhang J, Lu A, Li L, Yue J, Lu Y (2010). p16 modulates VEGF expression via its interaction with HIF-1alpha in breast cancer cells. Cancer Invest.

[45] Bedke J, Gauler T, Grünwald V, Hegele A, Herrmann E, Hinz S, Janssen J, Schmitz S, Schostak M, Tesch H, Zastrow S, Miller K (2017). Systemic therapy in metastatic renal cell carcinoma. World J Urol.

[46] Motzer RJ, Escudier B, McDermott DF, George S, Hammers HJ, Srinivas S, Tykodi SS, Sosman JA, Procopio G, Plimack ER, Castellano D, Choueiri TK, Gurney H, Donskov F, Bono P, Wagstaff J, Gauler TC, Ueda T, Tomita Y, Schutz FA, Kollmannsberger C, Larkin J, Ravaud A, Simon JS, Xu LA, Waxman IM, Sharma P, CheckMate I (2015). Nivolumab versus everolimus in advanced renal-cell carcinoma. N Engl J Med.

[47] Rosenberg JE, Hoffman-Censits J, Powles T, van der Heijden MS, Balar AV, Necchi A, Dawson N, O’Donnell PH, Balmanoukian A, Loriot Y, Srinivas S, Retz MM, Grivas P, Joseph RW, Galsky MD, Fleming MT, Petrylak DP, Perez-Gracia JL, Burris HA, Castellano D, Canil C, Bellmunt J, Bajorin D, Nickles D, Bourgon R, Frampton GM, Cui N, Mariathasan S, Abidoye O, Fine GD, Dreicer R (2016). Atezolizumab in patients with locally advanced and metastatic urothelial carcinoma who have progressed following treatment with platinum-based chemotherapy: a single-arm, multicentre, phase 2 trial. Lancet.

[48] Maia MC, Almeida L, Bergerot PG, Dizman N, Pal SK (2018). Relationship of tumor mutational burden (TMB) to immunotherapy response in metastatic renal cell carcinoma (mRCC). J Clin Oncol.

[49] Gerlinger M, Rowan AJ, Horswell S, Larkin J, Endesfelder D, Gronroos E, Martinez P, Matthews N, Stewart A, Tarpey P, Varela I, Phillimore B, Begum S, McDonald NQ, Butler A, Jones D, Raine K, Latimer C, Santos CR, Nohadani M, Eklund AC, Spencer-Dene B, Clark G, Pickering L, Stamp G, Gore M, Szallasi Z, Downward J, Futreal PA, Swanton C (2012). Intratumor heterogeneity and branched evolution revealed by multiregion sequencing. N Engl J Med.

[50] Soultati A, Stares M, Swanton C, Larkin J, Turajlic S (2015). How should clinicians address intratumour heterogeneity in clear cell renal cell carcinoma?. Curr Opin Urol.

[51] Sankin A, Hakimi AA, Mikkilineni N, Ostrovnaya I, Silk MT, Liang Y, Mano R, Chevinsky M, Motzer RJ, Solomon SB, Cheng EH, Durack JC, Coleman JA, Russo P, Hsieh JJ (2014). The impact of genetic heterogeneity on biomarker development in kidney cancer assessed by multiregional sampling. Cancer Med.

[52] Gerlinger M, Horswell S, Larkin J, Rowan AJ, Salm MP, Varela I, Fisher R, McGranahan N, Matthews N, Santos CR, Martinez P, Phillimore B, Begum S, Rabinowitz A, Spencer-Dene B, Gulati S, Bates PA, Stamp G, Pickering L, Gore M, Nicol DL, Hazell S, Futreal PA, Stewart A, Swanton C (2014). Genomic architecture and evolution of clear cell renal cell carcinomas defined by multiregion sequencing. Nat Genet.

[53] Knudson AG (1971). Mutation and cancer: statistical study of retinoblastoma. Proc Natl Acad Sci USA.

[54] Alexandrov LB, Nik-Zainal S, Wedge DC, Aparicio SA, Behjati S, Biankin AV, Bignell GR, Bolli N, Borg A, Borresen-Dale AL, Boyault S, Burkhardt B, Butler AP, Caldas C, Davies HR, Desmedt C, Eils R, Eyfjord JE, Foekens JA, Greaves M, Hosoda F, Hutter B, Ilicic T, Imbeaud S, Imielinski M, Jager N, Jones DT, Jones D, Knappskog S, Kool M, Lakhani SR, Lopez-Otin C, Martin S, Munshi NC, Nakamura H, Northcott PA, Pajic M, Papaemmanuil E, Paradiso A, Pearson JV, Puente XS, Raine K, Ramakrishna M, Richardson AL, Richter J, Rosenstiel P, Schlesner M, Schumacher TN, Span PN, Teague JW, Totoki Y, Tutt AN, Valdes-Mas R, van Buuren MM, van ‘t Veer L, Vincent-Salomon A, Waddell N, Yates LR, PedBrain I, Zucman-Rossi J, Futreal PA, McDermott U, Lichter P, Meyerson M, Grimmond SM, Siebert R, Campo E, Shibata T, Pfister SM, Campbell PJ, Stratton MR, Australian Pancreatic Cancer Genome I, Consortium IBC, Consortium IM-S (2013). Signatures of mutational processes in human cancer. Nature.

[55] Klatte T, Rossi SH, Stewart GD (2018). Prognostic factors and prognostic models for renal cell carcinoma: a literature review. World J Urol.

[56] Linehan WM, Spellman PT, Ricketts CJ, Creighton CJ, Fei SS, Davis C, Wheeler DA, Murray BA, Schmidt L, Vocke CD, Peto M, Al Mamun AA, Shinbrot E, Sethi A, Brooks S, Rathmell WK, Brooks AN, Hoadley KA, Robertson AG, Brooks D, Bowlby R, Sadeghi S, Shen H, Weisenberger DJ, Bootwalla M, Baylin SB, Laird PW, Cherniack AD, Saksena G, Haake S, Li J, Liang H, Lu Y, Mills GB, Akbani R, Leiserson MD, Raphael BJ, Anur P, Bottaro D, Albiges L, Barnabas N, Choueiri TK, Czerniak B, Godwin AK, Hakimi AA, Ho TH, Hsieh J, Ittmann M, Kim WY, Krishnan B, Merino MJ, Mills Shaw KR, Reuter VE, Reznik E, Shelley CS, Shuch B, Signoretti S, Srinivasan R, Tamboli P, Thomas G, Tickoo S, Burnett K, Crain D, Gardner J, Lau K, Mallery D, Morris S, Paulauskis JD, Penny RJ, Shelton C, Shelton WT, Sherman M, Thompson E, Yena P, Avedon MT, Bowen J, Gastier-Foster JM, Gerken M, Leraas KM, Lichtenberg TM, Ramirez NC, Santos T, Wise L, Zmuda E, Demchok JA, Felau I, Hutter CM, Sheth M, Sofia HJ, Tarnuzzer R, Wang Z, Yang L, Zenklusen JC, Zhang J, Ayala B, Baboud J, Chudamani S, Liu J, Lolla L, Naresh R, Pihl T, Sun Q, Wan Y, Wu Y, Ally A, Balasundaram M, Balu S, Beroukhim R, Bodenheimer T, Buhay C, Butterfield YS, Carlsen R, Carter SL, Chao H, Chuah E, Clarke A, Covington KR, Dahdouli M, Dewal N, Dhalla N, Doddapaneni HV, Drummond JA, Gabriel SB, Gibbs RA, Guin R, Hale W, Hawes A, Hayes DN, Holt RA, Hoyle AP, Jefferys SR, Jones SJ, Jones CD, Kalra D, Kovar C, Lewis L, Li J, Ma Y, Marra MA, Mayo M, Meng S, Meyerson M, Mieczkowski PA, Moore RA, Morton D, Mose LE, Mungall AJ, Muzny D, Parker JS, Perou CM, Roach J, Schein JE, Schumacher SE, Shi Y, Simons JV, Sipahimalani P, Skelly T, Soloway MG, Sougnez C, Tam A, Tan D, Thiessen N, Veluvolu U, Wang M, Wilkerson MD, Wong T, Wu J, Xi L, Zhou J, Bedford J, Chen F, Fu Y, Gerstein M, Haussler D, Kasaian K, Lai P, Ling S, Radenbaugh A, Van Den Berg D, Weinstein JN, Zhu J, Albert M, Alexopoulou I, Andersen JJ, Auman JT, Bartlett J, Bastacky S, Bergsten J, Blute ML, Boice L, Bollag RJ, Boyd J, Castle E, Chen YB, Cheville JC, Curley E, Davies B, DeVolk A, Dhir R, Dike L, Eckman J, Engel J, Harr J, Hrebinko R, Huang M, Huelsenbeck-Dill L, Iacocca M, Jacobs B, Lobis M, Maranchie JK, McMeekin S, Myers J, Nelson J, Parfitt J, Parwani A, Petrelli N, Rabeno B, Roy S, Salner AL, Slaton J, Stanton M, Thompson RH, Thorne L, Tucker K, Weinberger PM, Winemiller C, Zach LA, Zuna R, Cancer Genome Atlas Research N (2016). Comprehensive molecular characterization of papillary renal-cell carcinoma. N Engl J Med.

[57] Kauffman EC, Ricketts CJ, Rais-Bahrami S, Yang Y, Merino MJ, Bottaro DP, Srinivasan R, Linehan WM (2014). Molecular genetics and cellular features of TFE3 and TFEB fusion kidney cancers. Nat Rev Urol.

[58] Kovac M, Navas C, Horswell S, Salm M, Bardella C, Rowan A, Stares M, Castro-Giner F, Fisher R, de Bruin EC, Kovacova M, Gorman M, Makino S, Williams J, Jaeger E, Jones A, Howarth K, Larkin J, Pickering L, Gore M, Nicol DL, Hazell S, Stamp G, O’Brien T, Challacombe B, Matthews N, Phillimore B, Begum S, Rabinowitz A, Varela I, Chandra A, Horsfield C, Polson A, Tran M, Bhatt R, Terracciano L, Eppenberger-Castori S, Protheroe A, Maher E, El Bahrawy M, Fleming S, Ratcliffe P, Heinimann K, Swanton C, Tomlinson I (2015). Recurrent chromosomal gains and heterogeneous driver mutations characterise papillary renal cancer evolution. Nat Commun.

[59] Choueiri TK, Vaishampayan U, Rosenberg JE, Logan TF, Harzstark AL, Bukowski RM, Rini BI, Srinivas S, Stein MN, Adams LM, Ottesen LH, Laubscher KH, Sherman L, McDermott DF, Haas NB, Flaherty KT, Ross R, Eisenberg P, Meltzer PS, Merino MJ, Bottaro DP, Linehan WM, Srinivasan R (2013). Phase II and biomarker study of the dual MET/VEGFR2 inhibitor foretinib in patients with papillary renal cell carcinoma. J Clin Oncol.

[60] Davis CF, Ricketts CJ, Wang M, Yang L, Cherniack AD, Shen H, Buhay C, Kang H, Kim SC, Fahey CC, Hacker KE, Bhanot G, Gordenin DA, Chu A, Gunaratne PH, Biehl M, Seth S, Kaipparettu BA, Bristow CA, Donehower LA, Wallen EM, Smith AB, Tickoo SK, Tamboli P, Reuter V, Schmidt LS, Hsieh JJ, Choueiri TK, Hakimi AA, Chin L, Meyerson M, Kucherlapati R, Park WY, Robertson AG, Laird PW, Henske EP, Kwiatkowski DJ, Park PJ, Morgan M, Shuch B, Muzny D, Wheeler DA, Linehan WM, Gibbs RA, Rathmell WK, Creighton CJ, Cancer Genome Atlas Research N (2014). The somatic genomic landscape of chromophobe renal cell carcinoma. Cancer Cell.

[61] Joshi S, Tolkunov D, Aviv H, Hakimi AA, Yao M, Hsieh JJ, Ganesan S, Chan CS, White E (2015). The genomic landscape of renal oncocytoma identifies a metabolic barrier to tumorigenesis. Cell Rep.

[62] Sun M, Tong P, Kong W, Dong B, Huang Y, Park IY, Zhou L, Liu XD, Ding Z, Zhang X, Bai S, German P, Powell R, Wang Q, Tong X, Tannir NM, Matin SF, Rathmell WK, Fuller GN, McCutcheon IE, Walker CL, Wang J, Jonasch E (2017). HNF1B loss exacerbates the development of chromophobe renal cell carcinomas. Cancer Res.

[63] Casuscelli J, Weinhold N, Gundem G, Wang L, Zabor EC, Drill E, Wang PI, Nanjangud GJ, Redzematovic A, Nargund AM, Manley BJ, Arcila ME, Donin NM, Cheville JC, Thompson RH, Pantuck AJ, Russo P, Cheng EH, Lee W, Tickoo SK, Ostrovnaya I, Creighton CJ, Papaemmanuil E, Seshan VE, Hakimi AA, Hsieh JJ (2017). Genomic landscape and evolution of metastatic chromophobe renal cell carcinoma. JCI Insight.

[64] Hanahan D, Weinberg RA (2011). Hallmarks of cancer: the next generation. Cell.

